# A Distribution-Free Model for Longitudinal Metagenomic Count Data

**DOI:** 10.3390/genes13071183

**Published:** 2022-07-01

**Authors:** Dan Luo, Wenwei Liu, Tian Chen, Lingling An

**Affiliations:** 1 Department of Epidemiology and Biostatistics, The University of Arizona, Tucson, AZ 85721, USA; dluo4091@gmail.com; 2 Interdisciplinary Program of Statistics and Data Science, The University of Arizona, Tucson, AZ 85721, USA; wliu@email.arizona.edu; 3 Statistical and Quantitative Sciences, Takeda Pharmaceuticals, Cambridge, MA 02139, USA; tian.chen1@takeda.com; 4 Department of Biosystems Engineering, The University of Arizona, Tucson, AZ 85721, USA

**Keywords:** metagenomic, microbial, longitudinal, zero-inflated count model, correlation structure, distribution-free

## Abstract

Longitudinal metagenomics has been widely studied in the recent decade to provide valuable insight for understanding microbial dynamics. The correlation within each subject can be observed across repeated measurements. However, previous methods that assume independent correlation may suffer from incorrect inferences. In addition, methods that do account for intra-sample correlation may not be applicable for count data. We proposed a distribution-free approach, namely CorrZIDF, which extends the current method to model correlated zero-inflated metagenomic count data, offering a powerful and accurate solution for detecting significance features. This method can handle different working correlation structures without specifying each margin distribution of the count data. Through simulation studies, we have shown the robustness of CorrZIDF when selecting a working correlation structure for repeated measures studies to enhance the efficiency of estimation. We also compared four methods using two real datasets, and the new proposed method identified more unique features that were reported previously on the relevant research.

## 1. Introduction

With the advancement of high-throughput sequencing technologies, numerous time-course/longitudinal studies on microbiomes have been conducted [1,2,3,4,5]. By recording the temporal variation of microbial communities, this type of research can provide us insights into the stability of microbial communities and relationships among microbes. Detecting differentially abundant microbial features plays a critical role in population-based longitudinal studies, serving as potential biomarkers in biomedical research.

In metagenomic studies, the abundance of microbial taxa is characterized as counts. Due to the under-sampling of microbial samples, there may exist excess zeros for less abundant species. Early analysis methods fail to consider the unique characteristics of metagenomics data, which contain a large number of zeros due to the physical absence or under-sampling of the microbes [6,7]. Moreover, observations across different sampling points are correlated within each subject/patient. The independent correlation assumption may suffer from incorrect inferences.

Poisson-based log-linear models are widely used for modeling count data. The main property of those models is that the mean equals its variance. However, overdispersion exists universally, especially for metagenomic count data, in which the variance of the count is much larger than its mean. Thus, Poisson-based models yield a biased estimation for the parameters involved. The negative binomial (NB) method is more appropriate for modeling count data, since it allows an overdispersion estimation [8,9]. However, neither the Poisson nor the NB model can handle excess zeroes in the data, which cause the extra variability. The zero-inflated Poisson (ZIP) model and/or the zero-inflated negative binomial (ZINB) model are quite popular for modeling such zero-inflated count data, and assumes the data are from a mixture of a regular count distribution and a degenerate distribution at zero. The resulting proportion of zeros is the mixing probability of the two-component mixture distribution. However, the ZIP could still yield biased estimates when the non-zero counts in the data are overdispersed. Compared with the ZIP, the ZINB accounts for the overdispersion in the counts and can provide a more robust inference. However, the ZINB also yields biased estimates when overdispersion does not follow the negative binomial [10], since it is still based on a parametric model.

In the presence of overdispersion and excess zeros in the data, the generalized linear mixed-effect model (GLMM) can be used to describe random effects to account for correlated responses from repeated measurements over time. However, this approach lacks robustness when the data depart from the assumed distribution due to its parametric assumptions about random effect and response for inference. As a popular semi-parametric alternative, estimation equations only rely on the assumption of conditional mean response. For longitudinal studies, generalized estimating equations (GEE) are commonly used to address correlation among repeated response. However, either the ZIP or the ZINB is a mixture of two distributions, and simply modeling the mean response cannot identify the model parameters. Hall and Zhang [11] developed an approach for the ZIP and binomial data by integrating the maximum likelihood with GEE to deal with correlated longitudinal responses. However, this method still makes parametric assumptions for the response marginal distribution; thus, when the data deviates from the assumed marginal distribution, the performance of this method is affected. Dobbie and Welsh [12] developed a GEE approach for zero-inflated count data by modeling the mixture of zeros and truncated Poisson but it does not distinguish the zero mixture.

In order to overcome such difficulties, Chen and Li [13] proposed a two-part mixed-effect model (Zero-Inflated β Regression, ZIBR) for longitudinal microbiome compositional data using a logistic regression component to model presence/absence of a microbe in the samples. They then employed a β regression component with a random effect to model non-zero microbial abundance to account for the correlations among the repeated measurements on the same subject. However, this method is proposed for compositional data and assumes a β distribution for the non-zero data.

The fast zero-inflated negative binomial mixed modeling (FZINBMM) approach was propose by Zhang and Yi [14] to analyze and interpret the over-dispersed and zero-inflated longitudinal metagenomic count data. The FZINBMM approach is based on zero-inflated negative binomial mixed models (ZINBMMs) and employs a fast and stable EM-iterative weighted least-squares algorithm to fit the ZINBMMs. This model-fitting algorithm uses standard procedure of fitting linear mixed models, and can deal with many types of fixed and random effects and within-subject correlation structures.

A distribution-free functional response model (FRM) was proposed by Chen et al. [15] to model longitudinal zero-inflated count responses (noted as ZIDF in this paper) as a linear function of non-zero count responses and an identity function of the zero-count response. They extended the GEE model inference to general functions of FRM responses and focused on a working independence model.

The working correlation is often selected as independent (assuming no correlation across different observations/sampling points) or exchangeable (assuming all pairs of observations on the same subject have a common correlation) for convenience. Even so, the GEE estimation is consistent as the estimating equations are unbiased and the estimators of the regression parameter remain consistent for incorrect working structures. However, the exact form selected for a working correlation structure affects the efficiency. The efficiency of estimation will be increased when the correct correlation form is specified, particularly when the correlation within subjects is high [16,17,18,19]. However, misspecification of the working structure may result in a loss of efficiency in estimation of the regression parameter [20]. Moreover, the GEE that uses sandwich standard errors may suffer a higher type I error rate for small longitudinal designs with count outcomes [21].

Incorporating the working correlation structure into estimation can increase the relative efficiency of the estimation. In this paper, we extend the ZIDF to a longitudinal setting by introducing the working correlation structure estimation. The proposed method, shortened as CorrZIDF, is flexible such that it can handle different types of correlated structure without specifying the marginal distribution. In Section 2, we introduce the FRM for zero-inflated count responses and extend the model to account for correlation between time points. The application of CorrZIDF is demonstrated by simulation studies and real data analysis in Section 3. Finally, conclusions are drawn and discussed in Section 4.

## 2. Materials and Methods

### 2.1. Overview of Longitudinal Zero-Inflated Poisson (ZIP) and Zero-Inflated Negative Binomial (ZINB) Models

ZIP and ZINB models allow for overdispersion assuming two different types of subjects in the data: (1) those containing zero counts with a probability of 1 (i.e., True zero), and (2) those containing zero counts predicted by the standard Poisson/NB (i.e., structural zero). Observed zeros could be from either of these two types/groups; and if the zero is from the True zero group, it indicates that the observation is free from the probability of having a positive outcome [22]. Therefore, the overall model is a mixture of the probabilities from the two groups, which allows for both the overdispersion and excess zeros that cannot be predicted by the standard Poisson/NB model.

Let $Y_{ij}$ denote the longitudinal count response for subject i=1,…,N at time j where j=1,…,M. Under a longitudinal ZIP model, the distribution of $Y_{ij}$ is:

$$Y\sim \left\{\begin{array}{c} 0\\ Poisson\left(\mu_{ij}\right) \end{array}\right.\begin{array}{c} \mathrm{with}\;\mathrm{probability}\;\rho_{ij}\\ \;\mathrm{with}\;\mathrm{probability}\;1-\rho_{ij}, \end{array}$$

This is a degenerate distribution centered at 0 and a Poisson probability distribution function with mean $\mu_{ij}$. Then the probability distribution function can be written as

$$P\left(Y_{ij}=0|\;x_{ij}\right)=\rho_{ij}+\left(1-\rho_{ij}\right)e^{-\mu_{ij}}$$

$$P\left(Y_{ij}=y_{ij}|\;x_{ij}\right)=\left(1-\rho_{ij}\right)\frac{\mu_{ij}^{y_{ij}}e^{-\mu_{ij}}}{y_{ij}!},\;\mathrm{where}\;y_{ij}=1,2,\ldots$$

where the Poisson probability at 0 is modified by $\rho_{ij}+\left(1-\rho_{ij}\right)e^{-\mu_{ij}}$ to account for excess zeros, and $x_{ij}$ is a covariate.

In order to address overdispersed count response well within a group, the Poisson component can be replaced with the Negative Binomial distribution with parameters $\;(\rho_{ij},\;\mu_{ij},\;\tau )$ to form a ZINB model, where τ  accounts for the dispersion (for simplicity, assume a constant dispersion). Then under a ZINB model assumption the distribution of $Y_{ij}$ is

$$Y\sim \left\{\begin{array}{c} 0\\ NegativeBinomial\left(\mu_{ij},\tau \right) \end{array}\right.\begin{array}{c} \mathrm{with}\;\mathrm{probability}\;\rho_{ij}\\ \;\mathrm{with}\;\mathrm{probability}\;1-\rho_{ij} \end{array}$$

Then the probability distribution function can be written as

$$P\left(Y_{ij}=0|\;x_{ij}\right)=\rho_{ij}+\left(1-\rho_{ij}\right){\left(1+\tau \mu_{ij}\right)}^{\left(-1/\tau \right)}$$

$$P\left(Y_{ij}=y_{ij}|\;x_{ij}\right)=\left(1-\rho_{ij}\right)\frac{\Gamma \left(y_{ij}+\frac{1}{\tau }\right)}{\Gamma \left(y_{ij}+1\right)\Gamma \left(\frac{1}{\tau }\right)}\frac{{\left(\tau \mu_{ij}\right)}^{y_{ij}}}{{\left(1+\tau \mu_{ij}\right)}^{\left(y_{ij}+\frac{1}{\tau }\right)}},\mathrm{where}\;y_{ij}=1,2,\;\ldots$$

### 2.2. Functional Response Models (FRM) for Zero-Inflated Count Responses

Generally, for a cross-sectional study with N subjects at a specific point in time, we can write any zero inflated count model as:

$$y_{i}|x_{i}\;\sim \;\mathrm{Zero}-\mathrm{inflated}\;\mathrm{Distribution}\;\left(\rho_{i},\;\;\mu_{i}\right)$$

where $\rho_{i}$, the proportion of zeros, can be estimated through a logit link in a regression model, logit($\rho_{i}$) = $u_{i}^{T}\beta_{u}$; and $\mu_{i}$, the mean response, can be estimated through a log link in a regression model log ($\mu_{i}$) = $v_{i}^{T}\beta_{v}$, where $u_{i}$ and $v_{i}$ are two subsets of covariates $x_{i}$, and $\beta ={\left(\beta_{u},\beta_{v}\right)}^{T}.$ This equation can be extended to any zero-inflated count model, e.g., ZIP and ZINB.

Under a cross-sectional setting, the conditional variance of the count response under ZINB for the degenerate distribution centered at 0 is $\mathrm{Var}\left(y_{i}|x_{i}\right)=\mu_{i}\left(1+\frac{\;\mu_{i}}{\tau }\right)$, which is larger than the conditional mean, $E\left(y_{i}|x_{i}\right)=\mu_{i}$. For the Moment-based model, the inference is valid regardless of whether $y_{i}$ given $x_{i}$ follows Poisson, NB, or any other distribution as long as log ($\mu_{i}$) = $x_{i}^{T}\beta \;$ is a correct model for the conditional mean [23,24]. Unfortunately, modeling the mean parameter alone in ZIP or ZINB is not able to estimate $\beta_{u}$ and $\beta_{v}$, since the mean alone is not sufficient to identify those parameters.

Tang et al. [25] proposed a nonparametric FRM approach to model the count responses through two functions, $f_{1i}=I\left(y_{i}=0\right)$ and $f_{2i}=y_{i}\;(\mathrm{where}\;y_{i}>0)$, to describe the model parameters. This method has been proved to be robust for a broader class of dispersion for cross-sectional data, such as overdispersion under ZIP, ZINB, or normal random effects. Under this approach, the expected value of $y_{i}$ can be decomposed as:

$$E\;(y_{i})=E{\left(f_{1i},\;f_{2i}\right)}^{T}={\left(h_{1i},\;h_{2i}\right)}^{T},$$

where $h_{1i}={\mathrm{logit}}^{-1}\left(u_{i}^{T}\beta_{u}\right)+\frac{\exp\left(-\exp\left(v_{i}^{T}\beta_{v}\right)\right)}{1+\exp\left(u_{i}^{T}\beta_{u}\right)},h_{2i}=\frac{\exp\left(v_{i}^{T}\beta_{v}\right)}{1+\exp\left(u_{i}^{T}\beta_{u}\right)}.$

Such distribution-free regression models are defined as functional response models (FRM).

Under the longitudinal setting with M observations/sampling points, we may use a parametric modeling approach to model $y_{ij}$ as a function of $x_{ij}$, for instance, generalized linear mixed-effect models (GLMM), which can account for correlation from repeated sampling. However, the parametric models suffer from interpretational and computational issues when the observed data depart from the assumed distribution. Generalized estimating equations (GEE) is a widely-used distribution-free alternative with inference based on the GEE specified the conditional mean of $y_{ij}$ given $x_{ij}$. For traditional longitudinal data (i.e., without zero-inflation issues), GEE provides a robust estimation for addressing overdispersed count responses. However, for zero-inflated longitudinal models that assume a two-part mixture (i.e., zero and non-zero parts), GEE cannot work well as it does not provide sufficient information for all parameters in a mixture model setting, since only modeling the mean response provides insufficient information to estimate the parameters in the two-part model.

Chen et al. [15] proposed a zero-inflated distribution-free approach (we term it ZIDF) to extend the FRM model to the longitudinal setting by considering longitudinal responses across M sampling/time points. Let $y_{ij},x_{ij}$, $u_{ij}$ and $v_{ij}$ denote the respective variables at time *j* (1 ≤j≤M), the FRM can be written as:

$$f_{ij}={\left(f_{1ij},\;f_{2ij}\right)}^{T},\;\;h_{ij}={\left(h_{1ij},\;h_{2ij}\right)}^{T},\;f_{1ij}=I\left(y_{ij}=0\right),\;f_{2ij}=y_{ij},$$

$$h_{1ij}=\rho_{ij}+\left(1-\rho_{ij}\right)\exp\left(-\mu_{ij}\right)={\mathrm{logit}}^{-1}\left(u_{ij}^{T}\beta_{u}\right)+\frac{\exp\left(-\exp\left(v_{ij}^{T}\beta_{v}\right)\right)}{1+\exp\left(u_{ij}^{T}\beta_{u}\right)},$$

$$h_{2ij}=\left(1-\rho_{ij}\right)\mu_{ij}=\frac{\exp\left(v_{ij}^{T}\beta_{v}\right)}{1+\exp\left(u_{ij}^{T}\beta_{u}\right)},$$

$$\mathrm{Var}\left(f_{1ij}\right)=h_{1ij}\left(1-h_{1ij}\right),$$

$$\mathrm{Var}\left(f_{2ij}\right)=\mu_{ij}\left(1+\rho_{ij}\mu_{ij}\right)\left(1-\mu_{ij}\right),\mathrm{where}\;1\leq i\leq N,1\leq j\leq M.$$

Note that the mathematical notations in bold here represent corresponding vectors. The inference for this model will be discussed in the next section.

### 2.3. FRM Model Inference

Following Chen et al. [15], let $\beta ={\left(\beta_{u}^{T},\beta_{v}^{T}\right)}^{T}$ and $f_{i}={\left(f_{i1}^{T},f_{i2}^{T},\ldots ,f_{iM}^{T}\right)}^{T}$, $h_{i}={\left(h_{i1}^{T},h_{i2}^{T},\ldots ,h_{iM}^{T}\right)}^{T}$, and define the following function as $D_{i}=\frac{\partial }{\partial \beta }h_{i}$*,* $S_{i}=f_{i}-h_{i}$. β can be estimated by solving the following GEE set:

$$U_{N}\left(\beta \right)=\overset{N}{\underset{i=1}{\sum }}U_{Ni}\left(\beta \right)=\overset{N}{\underset{i=1}{\sum }}D_{i}V_{i}^{-1}S_{i}=0.$$

$V_{i}$, a matrix function of $x_{ij}$, reflects the correlation between the $f_{ij}\;$ over time, where

$$V_{i}=A_{i}^{\frac{1}{2}}R\left(\alpha \right)A_{i}^{\frac{1}{2}},\;A_{i}={\mathrm{diag}}_{j}\left(A_{ij}\right),\;A_{ij}=\mathrm{Var}\left(f_{ij}|x_{ij}\right).$$

$R\left(\alpha \right)$ is the working correlation matrix parameterized by α among the components of $f_{i}$. By substituting an estimate $\hat{\alpha }$ in place of α, it can be solved for β. If $\hat{\alpha }$ is $\sqrt{n}$-consistent, the GEE estimate $\hat{\beta }$ obtained by solving above is consistent and asymptotically normal with

$$\sqrt{n(}\hat{\beta }-\beta )\to_{d}N\left(0,\Sigma_{\beta }\right),\;\Sigma_{\beta }=B^{-1}E\left(D_{i}V_{i}^{-1}S_{i}S_{i}^{T}V_{i}^{-1}D_{i}^{T}\right)B^{-T},\;\mathrm{where}\;B=E\left(D_{i}V_{i}^{-1}D_{i}^{T}\right)$$

$\to_{d}\;$ means that the distribution is converged [23]. $\Sigma_{\beta }$ is consistently estimated by substituting moment estimates with the following respective parameters:

$$\hat{\Sigma_{\beta }}={\hat{B}}^{-1}\left(\frac{1}{N}\overset{N}{\underset{i=1}{\sum }}\hat{D_{i}}{\hat{V}}_{i}^{-1}{\hat{S}}_{i}{\hat{S}}_{i}^{T}{\hat{V}}_{i}^{-1}{\hat{D}}_{i}^{T}\right){\hat{B}}^{-T},\;\mathrm{where}\hat{\;B}=\frac{1}{N}\overset{N}{\underset{i=1}{\sum }}\hat{D_{i}}{\hat{V}}_{i}^{-1}{\hat{D}}_{i}^{T}$$

The simplest choice for $R\left(\alpha \right)\;$ is the working independence model $R\left(\alpha \right)=I_{2M}.$ However, the GEE estimation may not be consistent when the data has time-varying covariates that follow some working correlation structures. Moreover, such a simple working independence model may incur loss of efficiency in parameter estimation.

The First-order linear autoregressive (AR (1)) is a common correlation structure for longitudinal data, where the correlation between two adjacent time points is a constant. For a longitudinal design that consists of *N* subjects, for each subject $\left(i=1,2,\ldots ,\;N\right)$*,* there are M observations (assume the number of observations for each subject remains the same) and $Y_{ij}$ denotes the $j^{th}$ response. The moment correlation between two observations can be noted as:

$$\mathrm{Corr}\left(Y_{ij},Y_{i,j+h}\right)=\alpha^{h},\;h=0,1,2,\ldots ,M-j$$

The correlation matrix is written as:

$$\left(\begin{array}{cccc} 1 & \alpha & \cdots & \alpha^{M-1}\\ \alpha & 1 & \cdots & \vdots \\ \vdots & \vdots & \ddots & \alpha \\ \alpha^{M-1} & \ldots & \alpha & 1 \end{array}\right)$$

where $\;\hat{\alpha }=\frac{1}{\left(K-2\right)}\sum_{i=1}^{N}\sum_{j\leq M-1}e_{ij}e_{i,j+1},$ where $K=\sum_{i=1}^{N}\left(M-2\right)$, and Pearson residuals $e_{ij}\;\mathrm{can}\;\mathrm{be}\;\mathrm{estimated}\;\mathrm{as}\;\left(Y_{ij}-E(Y_{ij}|X_{ij}\right))/\sqrt{\mathrm{Var}\left(Y_{ij}|X_{ij}\right)}$ (here we only include the intercept and treatment effect for covariate X).

Consider the AR (1) correlation structure for the zero-inflated data as following. In this paper, for each subject, we propose a new method (CorrZIDF) to estimate the correlation α using the modified bivariate Pearson residuals as:

$$R\left(\alpha \right)=\left(\begin{array}{cccc} I_{2} & \alpha J_{2} & \cdots & \alpha^{M-1}J_{2}\\ \alpha J_{2} & I_{2} & \cdots & \vdots \\ \vdots & \vdots & \ddots & \alpha J_{2}\\ \alpha^{M-1}J_{2} & \ldots & \alpha J_{2} & I_{2} \end{array}\right)$$

$$I_{2}=\left(\begin{array}{cc} 1 & 0\\ 0 & 1 \end{array}\right),{\;J}_{2}=\left(\begin{array}{cc} 1 & 1\\ 1 & 1 \end{array}\right),\;0<\alpha <1$$

$$e_{ij}^{T}e_{ij}=\frac{{\left(f_{ij}-h_{ij}\left(\beta \right)\right)}^{T}\left(f_{ij}-h_{ij}\left(\beta \right)\right)}{\mathrm{Var}\left(f_{ij}\right)},$$

$\mathrm{where}\;e_{ij}={\left(e_{1ij},e_{2ij}\right)}^{T}$, then $\hat{\alpha }$ can be estimated as:

$$\hat{\alpha }=\frac{1}{{\left(K-2\right)}^{2}}\overset{N}{\underset{i=1}{\sum }}\underset{j\leq M-1}{\sum }\left(e_{1ij}e_{1i,j+1}+e_{2ij}e_{2i,j+1}\right),\;\mathrm{where}\;K=N\left(M-2\right).$$

CorrZIDF is also implemented with exchangeable correlation structure estimation, which assumes all pairs of observations on the same subject share a common correlation. For the zero-inflated data, the correlation structure can be written as:


$$R\left(\alpha \right)=\left(\begin{array}{cccc} I_{2} & \alpha J_{2} & \cdots & \alpha J_{2}\\ \alpha J_{2} & I_{2} & \cdots & \vdots \\ \vdots & \vdots & \ddots & \alpha J_{2}\\ \alpha J_{2} & \ldots & \alpha J_{2} & I_{2} \end{array}\right)$$

Then we propose the following estimation as

$$\hat{\alpha }=\frac{1}{{\left(K-2\right)}^{2}}\overset{N}{\underset{i=1}{\sum }}\underset{j\neq l}{\sum }\left(e_{1ij}e_{1il}+e_{2ij}e_{2il}\right),\;\mathrm{where}\;K=NM\left(M-2\right).$$

Here we focus on testing the effect from the non-zero part. The significance for the non-zero parameter $\beta_{v}$ for each feature is assessed using the Wald test and the *p*-values are adjusted with the Benjamini–Hochberg (BH) procedure [26] to control the false discovery rate (FDR).

### 2.4. Simulation Setting

A series of simulated metagenomic studies were conducted to evaluate the performance of CorrZIDF, and to compare it to ZIDF, ZIBR, and FZINBMM by using the Copula method. Copula is a joint cumulative distribution function of a multiple dimensional vector [27]. Given the fact that, by its probability integral transformation, any continuous random variable can be transformed to be uniformly distributed over the interval (0, 1), copulas can be used to provide a multivariate dependence structure separately from the marginal distribution [27,28]. Copula package in R is used to name the marginal distribution of each vector and set the correlation among the vectors. We used elliptical copulas in this package due to its easy implementation. The copula has a dispersion matrix and after standardization it becomes correlation matrix that determines the dependence structure. Commonly used dependence structures in this package include autoregressive of order 1 (AR (1)) and exchangeable.

The data were simulated under a zero-inflated Poisson distribution, where the zero percentage was modeled to be negatively correlated to the means. That is, the zero-percentage decreases as the mean count value increases.

Two groups/conditions (treatment vs. control) of data were simulated. For the treatment group, we simulated a linear increasing pattern of microbial abundance for differential abundant features (DAFs); for the control group, the features were assumed to be static or stable over time. The rest of the features are assumed to have the same patterns along time for both conditions. Two levels of correlation (ρ= 0.6 and ρ = 0.9) were examined to evaluate the model performance under both correlation structures (i.e., AR (1) and exchangeable). In addition, the counts within each time point were also generated to mimic an exponential growth pattern for microbes.

For each combination of parameter settings (i.e., groups of microbes with a certain correlation structure and a certain correlation level, under a certain microbial growth pattern, and at a certain sample size), we simulated 20 datasets, each consisting of 1000 features/species over 10 sampling/time points; 200 features were assumed to have differential abundance, noted as differentially abundant features, and the remaining 800 features were simulated to be stable over time, noted as non-differentially abundant features. Two levels of sample size were also compared. The details of the simulation settings are shown in Table 1. The data were simulated using the Copula method.

## 3. Results

### 3.1. Simulation Results

**Figure 1:**
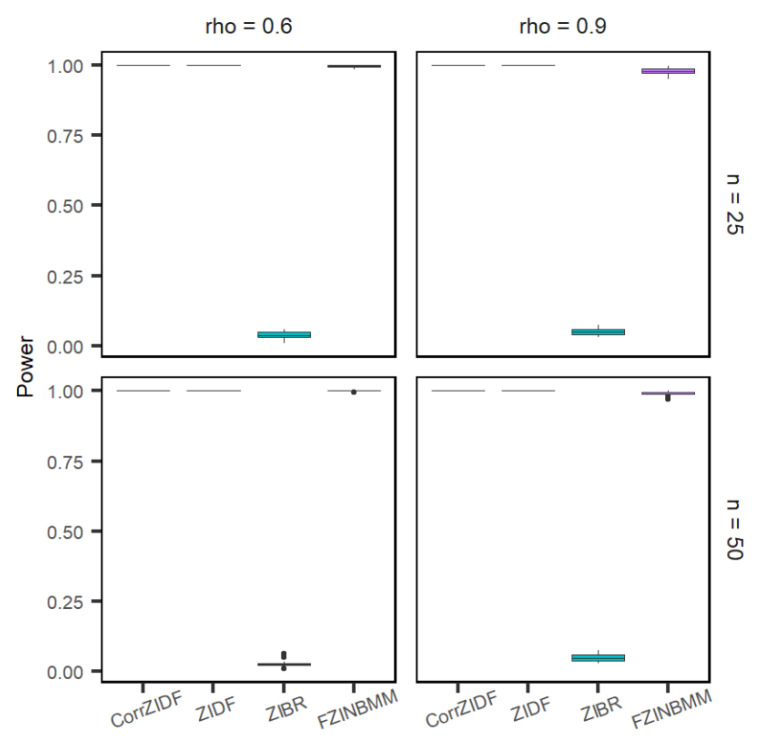
Boxplots for the power under various settings based on 20 replicated simulations with 1000 features (including 200 DAFs) after adjusting multiple comparisons. The *p*-values are adjusted by the BH procedure. Assume AR (1) correlation structure across different sampling points.

**Figure 2:**
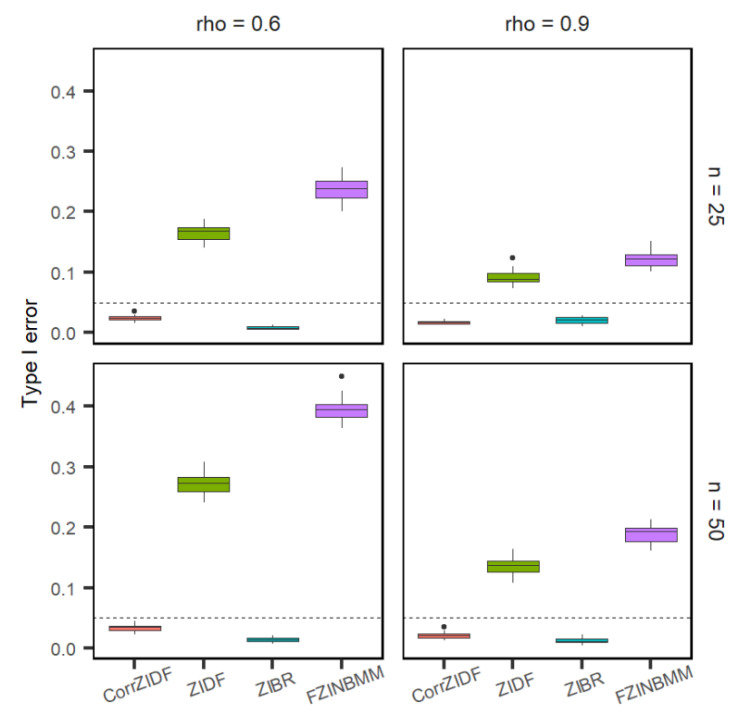
Boxplots of Type I error rates under various settings based on 20 replicated simulations with 1000 features (including 200 DAFs) after adjusting multiple comparisons. The dashed line represents the cutoff of 0.05. Assume AR (1) correlation structure across different sampling points. The *p*-values are adjusted by the BH procedure.

**Figure 3:**
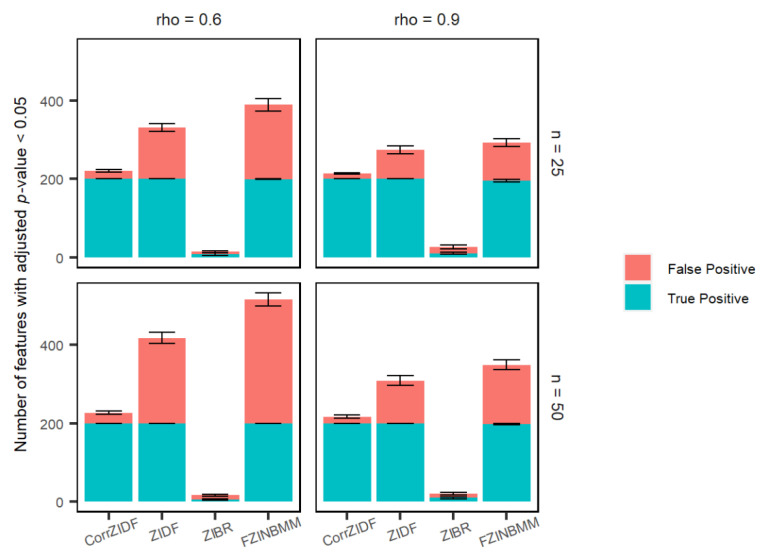
Bar plots of the numbers of detected true and false positives under various settings with 1000 features and 20 replicated simulations. Each bar represents the total number of features that are detected as statistically significant post BH adjustment, and the short error bars represent the standard deviation from 20 replications. Note: the true number of DAFs in the simulation is 200. Assume AR (1) correlation structure across different sampling points.

The comparison of the CorrZIDF to the existing methods, ZIDF, ZIBR and FZINBMM, was conducted on the simulated count data. The performance metrics include false positive rate (FPR) or type I error, and true positive rate (TPR) or power. Figure 1, Figure 2 and Figure 3 show the results that each marginal follows the AR (1) correlation structure across different sampling points. The results show that the CorrZIDF greatly outperforms the other methods under all scenarios in terms of different comparisons.

The power plot for the cutoff of 0.05 is shown in Figure 1. The type I error plot on adjusted *p*-value is shown in Figure 2. The ZIDF performs with higher power but with a substantially inflated type I error rate. The ZIBR controls the type I error well but has little power to detect changing features, which implies that the method is too conservative. The FZINBMM also performs with higher power, but the type I error rate is the worst among the four methods. Compared to the existing methods, the CorrZIDF presents both a well-controlled type I error and a consistently higher power across different simulation settings. When the correlation is higher, the CorrZIDF, ZIDF and FZINBMM show better performance in terms of lowering the type I error, as the changing pattern is more consistent due to the sampling points being more correlated. However, as the sample size increases, ZIDF and FZINBMM will detect more false signals, resulting in an inflated type I error. By contrast, ZIBR shows a lower type I error as the sample size increases; however, its power remains quite low due to its conservative nature.

In Figure 3, it is clear that the CorrZIDF, ZIDF and FZINBMM have similar numbers of true positive features, and the CorrZIDF is the one with the lowest number of false positive features among these three methods. Even though the ZIBR has the lowest number of false positive features overall, it has the worst performance detecting true positive features.

The results when each margin follows the exchangeable correlation structure across different samples are shown in the Supplemental Figures S1–S3. Similarly, the CorrZIDF greatly outperforms the other methods under all scenarios in terms of different comparisons. In all settings in Figure S1, the CorrZIDF and ZIDF have similar performance in the power and their performances are the best among the four methods. The FZINBMM performs slightly worse than the above two methods. The ZIBR remains the lowest power due to its conservative nature. The type I error plots in Figure S2 show that the ZIDF has the highest type I error among the four methods. When the correlation is higher, the CorrZIDF, ZIDF and FZINBMM show better performance. The ZIBR in n = 25 is the only one that performs a little bit worse when the correlation increases. The FZINBMM is the method that improves the performance greatly. As the sample size increases, the ZIDF and FZINBMM will detect more false signals, resulting in an inflated type I error. In Figure S3, not surprisingly, the ZIBR can only detect a small number of true positive features. The CorrZIDF, ZIDF and FZINBMM can capture a similar number of true positives, while the CorrZIDF can remain a small number of false positive features consistently across all settings.

An additional simulation study with a smaller correlation level (ρ=0.3) and smaller sample sizes (five and ten subjects per condition) in AR (1) correlation structure are examined as well, due to the fact of many real datasets are usually with small numbers of subjects. The results are shown in Supplemental Figures S4–S6. For the power in Figure S4, in all settings, the CorrZIDF and ZIDF have a similar performance in the power and their performances are the best among the four methods. The FZINBMM has less power when the correlation level or number of subject decreases. The ZIBR remains the lowest power in all settings due to its conservative nature. For the type I error in Figure S5, it is controlled very well by the ZIBR, while it is inflated consistently across all settings in the ZIDF. The FZINBMM sometimes inflates type I error, and the CorrZIDF controls the type I error in almost all settings. In Figure S6, the ZIBR only can catch a few true positives, and the FZINBMM misses true positives for some settings in smaller sample size. The CorrZIDF and ZIDF perform similarly in terms of detecting true positives and they can capture almost all true positives, but the CorrZIDF contains much smaller false positives consistently across all settings.

We also compared the method’s performance with our previously proposed method, the metaDprof, a spline-based method to detect differentially abundant features based on permutation tests [29]. Based on the simulation results, the metaDprof controls type I error well and shows comparable power (results not shown), but the metaDprof is substantially computationally costly.

To complete testing each method on a simulation dataset with 1000 features across 10 sampling points with 25 samples using two CPUs, the CorrZIDF took 10 min, the ZIDF took 5 min, the ZIBR used 2.5 h and the FZINBMM took about 8 min; however, the metaDprof needed 2 h and 10 min with 168 CPUs.

### 3.2. Real Data Analysis

We applied all four methods to two real datasets, a pregnancy study and a humanized gnotobiotic mouse gut study, and the results are shown in the following sections.

#### 3.2.1. Pregnancy Study

In a case-control study of 40 pregnant women, 7 of them delivered preterm (before gestational week 37), 5 marginal (gestational week 37) and 28 delivered at term (>37 gestational weeks) [30]. From 40 of these women, a bacterial taxonomic composition of 3767 specimens was collected prospectively and weekly during gestation and monthly after delivery from the vagina, distal gut, saliva and tooth/gum. Five preterm women who had ten consecutive weeks of vaginal measurements before delivery were chosen for analysis. To have a balanced design, we selected five women who had ten consecutive weeks of vaginal measurements before delivery from the term group. The microbiome data was aggregated to genus level and there were 45 genera left for downstream analysis.

**Figure 4:**
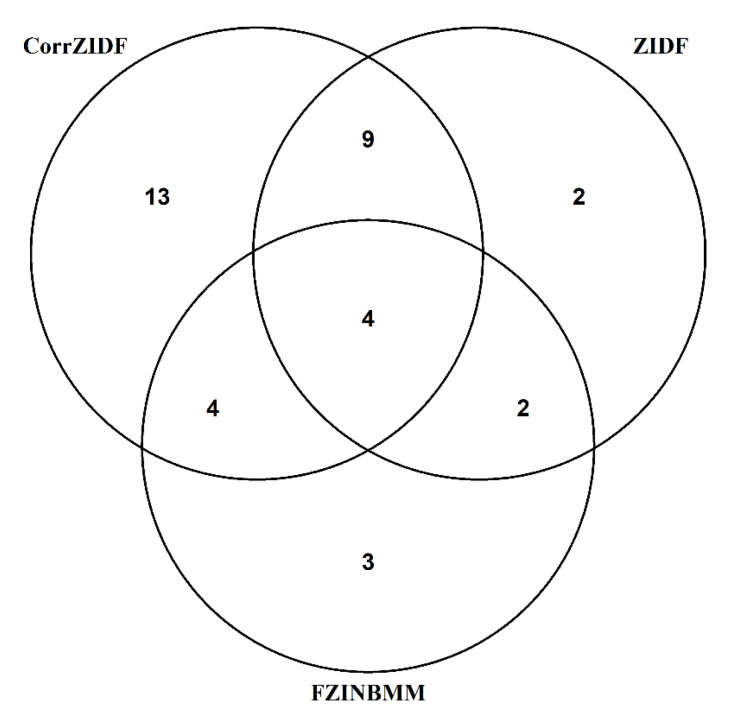
A Venn diagram of the distribution of the significantly differentiated results by the CorrZIDF, ZIDF and FZINBMM for a pregnancy dataset.

Among 45 genera, there were 30 genera that showed significantly differentiated results by the CorrZIDF, 17 genera by the ZIDF, 13 genera by the FZINBMM, and there were no significantly differentiated genus by the ZIBR. The distribution of these significantly differentiated genera in each method are shown in a Venn diagram in Figure 4. There were four genera captured by all three methods. Each method also captured a certain number of unique genera, i.e., 13 unique ones by the CorrZIDF, 2 by the ZIDF and 3 by the FZINBMM, respectively. To compare the performance of the methods, we focus on the unique genera listed in Table 2. All of them have been reported in the literature of preterm delivery-related studies. The details of relevance can be found in Table 2.

#### 3.2.2. Humanized Gnotobiotic Mouse Gut Study

Another real dataset we used to compare our proposed approach with other methods was from a humanized gnotobiotic mouse gut study with two groups of six germ-free adult male C57BL/6J mice feeding on a low-fat diet (plant polysaccharide-rich diet) and a Western diet (high-fat and high-sugar diet) [51]. Each mouse’s fecal sample went through PCR amplification of the bacterial 16S rRNA gene V2 weekly during an 8-week period. After aggregating the OTU count data to the genus level and basic filtering, there were 30 genera left for downstream analysis.

**Figure 5:**
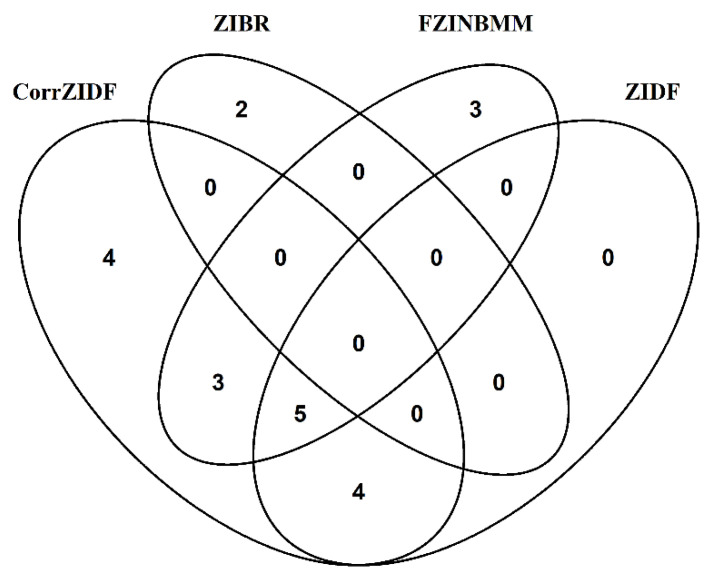
A Venn diagram of the distribution of the significantly differentiated results in the CorrZIDF, ZIDF, ZIBR and FZINBMM in diet dataset.

Among these genera, there were a total of 21 genera showing significantly differentiated results; among them, 16 were detected by the CorrZIDF. The distribution of these significantly differentiated genera in each method were shown in a Venn diagram (Figure 5). There were no overlapping genera captured by all four methods. Each method also captured the different number of unique genera, four unique genera by the CorrZIDF, two genera by the ZIBR, and three genera by the FZINBMM. To compare the performance of the methods, we focus on the unique genera, listed in Table 3. All of them have been reported in the diet-related literature. The details of the relevance can be found in Table 3. As 16 out of 21 genera are detected by the CorrZIDF, the new method shows the most power in analyzing the mouse gut dataset.

## 4. Discussion

With the advent of high throughput sequencing and analytical tools, longitudinal studies provide increased insights into the stability of microbial communities and relationships among microbes. Most existing methods use either a parametric method by including a random effect to account for the correlations among repeated measurements on the same subject, or a nonparametric model without specifying the correlation structure. However, modeling the count data through inappropriate statistical distributions or ignoring the correlation across time would incur an incorrect estimation.

We extended the ZIDF, the nonparametric model, by accounting for the correlation among repeated measurements. The ZIDF utilized a nonparametric zero-inflated count model that does not need assumptions about each margin under a longitudinal setting. However, the method assumes an independent correlation across different samples over time. Even though their method has higher power to detect significant features, it incurs a larger type I error. The ZIBR shows a well-controlled type I error across different scenarios; however, it shows the lowest power in detecting significant features. The ZIBR is proposed to analyze compositional data, which may explain its loss in power when we convert the count data to compositional data in order to apply this method. In addition, it assumes that the compositional data follow a β distribution, which may not be true. Generally, the FZINBMM shows a higher type I error than the CorriZIDF, with a comparable power. Our proposed method, the CorrZIDF, extending the ZIDF, shows a robust superior performance under various scenarios (i.e., different margin distributions and different correlation structures).

This project focused on testing the effect on non-zero counts from the mixture; the method can also provide parametric estimation on the zero-count portion of the data, especially when researchers are interested in estimating biomarkers’ effects for the always-zero group. Currently, most of the association testing approach focuses on independent subjects within each sampling community. However, in a real-world setting, some microbial species are correlated under different environments or medical treatments. For a future study, we will extend the CorrZIDF to account for such correlation between different features within a sample to better understand and utilize information about the microbial dynamic. With these potential biomarkers, scientists may utilize such information to target screening in order to better understand the biological dynamics and its association with treatments/covariates.

## Figures and Tables

**Table 1:** Summary of parameter settings for the simulation studies. Two correlation structures, AR (1) and exchangeable were generated.

Setting	AR (1)		Exchangeable	
25 subjects per condition	Moderately Correlated ρ=0.6	Highly Correlated *ρ* =0.9	Moderately Correlated *ρ* =0.6	Highly Correlated ρ=0.9
50 subjects per condition				

**Table 2:** List of unique genera by each method for the pregnancy data.

Method	Genus	Relevance	Reference
CorrZIDF	Acinetobacter	Acinetobacter infection in adverse pregnancy and perinatal outcomes	[31,32]
	Aerococcus	Low abundance in preterms	[33]
	Atopobium	High relative abundance of Atopobium vaginae at the midtrimester was highly predictive of preterm birth	[34]
	Bacteroides	Abundance reduction in Bacteroides in women who delivered preterm	[35,36]
	Brevibacterium	Occasionally found in the placenta, considered as contaminants	[37]
	Campylobacter	Associated with an increased risk of spontaneous abortion, stillbirth, and preterm delivery	[38]
	Fusobacterium	Associated with preterm birth and has been isolated from the amniotic fluid, placenta, and chorioamnionic membranes of women delivering prematurely	[39]
	Mobiluncus	For women with a prior preterm delivery, high level of Mobiluncus significantly indicate a spontaneous preterm delivery	[40]
	Oligella	Mostly found as a commensal organism of the human genitourinary tract, which is also the main infection site	[41]
	Peptostreptococcus	Pregnant women with Bacterial vaginosis including *Peptostreptococcus* and other bacteria have increased risk of preterm labor and preterm premature rupture of membranes.	[42]
	Porphyromonas	Significantly high abundance in preterms	[43]
	Sneathia	Low abundance found in preterm	[33]
	Sutterella	Associated with metabolic/inflammatory variables across pregnancy in Gestational diabetes mellitus patients; hyperglycemia in the second and third trimester of pregnancy is an independent risk factor and a better predictor of prematurity.	[44,45]
ZIDF	Facklamia	More abundant in animals that failed to establish a pregnancy	[46]
	Ureaplasma	High abundance of *Ureaplasma* is associated with preterm birth	[30,47]
FZINBMM	Actinomyces	Actinomyces infections in pregnancy are rare but, if they occur, have been linked primarily with preterm deliveries.	[48]
	Anaerococcus	The vaginal microbiota of Non-aboriginal women had higher relative abundance of the taxa Anaerococcus	[49]
	Finegoldia	Associated with bacterial vaginosis, which is linked to an increased risk of preterm birth:	[50]

**Table 3:** List of unique genera by each method for the mouse diet data.

Method	Genus	Relevance	Reference
CorrZIDF	Anaerofilum	The relative abundances of Anaerofilum were significantly lower in the obese group.	[52]
	Bilophila	Increased abundance of Bilophila has been associated with fat feeding and inflammation	[53]
	Clostridium	High fat diet lowers C. butyricum levels; C. butyricum maybe one of the species that constitute a core microbiota involved in energy storage and metabolism through mechanisms that are not yet known; Clostridium XIVb is more abundant in high fat diet group than the control group.	[54,55]
	Eggerthella	It metabolized amino acids rather than sugar	[55]
ZIBR	Akkermansia	Akkermansia muciniphila abundance was strongly and negatively affected by high-fat diet feeding	[56]
	ErysipelotrichaceaeIncertaeSedis	Aaccelerated postnatal growth suppressed the abundance of Erysipelotrichaceae_incertae_sedi	[55]
FZINBMM	Alistipes	Were significantly different between the high-fat diet and low-fat diet groups	[57]
	Bryantella	Relatively high abundance in the gut in high protein fed mice	[58]
	Mogibacterium	In overweight people, Mogibacterium is associated with PUFA-rich (polyunsaturated fatty acid) diets	[59]

## Data Availability

The OTU count tables for pregnancy data is available at [https://susan.su.domains/papers/PNASRR.html (accessed on 1 June 2022)]. The metagenomic count dataset was downloaded from metagenomeSeq R package [60]. R code to implement CorrZIDF method can be downloaded from github.com/anlingUA/CorrZIDF (accessed on 20 June 2022).

## Supplementary Materials

The following supporting information can be downloaded at: https://www.mdpi.com/article/10.3390/genes13071183/s1, Figure S1: Boxplots for the power under exchangeable correlation structure, Figure S2: Boxplots of Type I error rates under exchangeable correlation structure, Figure S3: Bar plots of the numbers of detected true and false positives under exchangeable correlation structure. Figure S4: Boxplots of the power for small number of subjects. Figure S5: Boxplots of Type I error rates for small number of subjects. Figure S6. Bar plots of the numbers of detected true and false positives for small number of subjects.
