# Supplementary material for: A Distribution-Free Model for Longitudinal Metagenomic Count Data
Source: Genes (Basel). 2022 Jul 1;13(7):1183. doi: 10.3390/genes13071183 (PMC9316307; doi:10.3390/genes13071183)
Supplement: Supplementary file 1 [file genes-13-01183-s001.zip › genes-1744147-supplementary.pdf]

Supplementary File

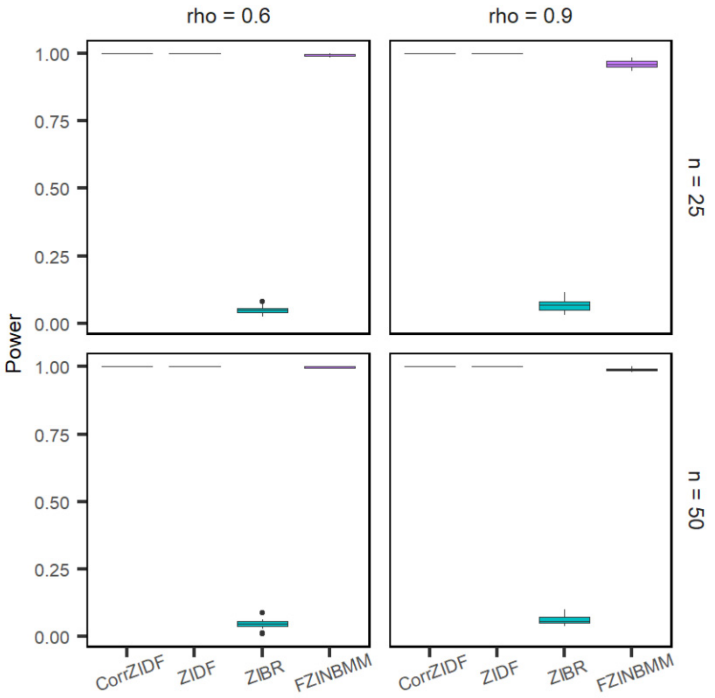

Figure S1. Boxplots for the power under exchangeable correlation structure. Boxplots in 4 (25/50 subjects per condition with moderate/high correlation) settings based on 20 replicated simulations with 1000 features (including 200 DAFs) after adjusting multiple comparisons by the BH procedure.

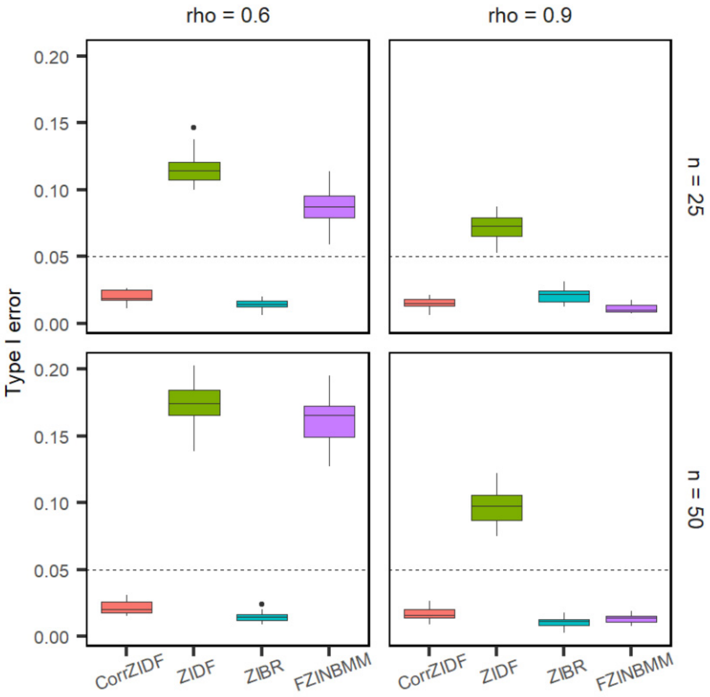

Figure S2. Boxplots of Type I error rates under exchangeable correlation structure. Boxplots in 4 (25/50 subjects per condition with moderate/high correlation) settings based on 20 replicated simulations with 1000 features (including 200 DAFs) after adjusting multiple comparisons by the BH procedure. The dashed line represents the cutoff of 0.05. CorrZIDF and ZIBR control the type I error well in all 4 settings.

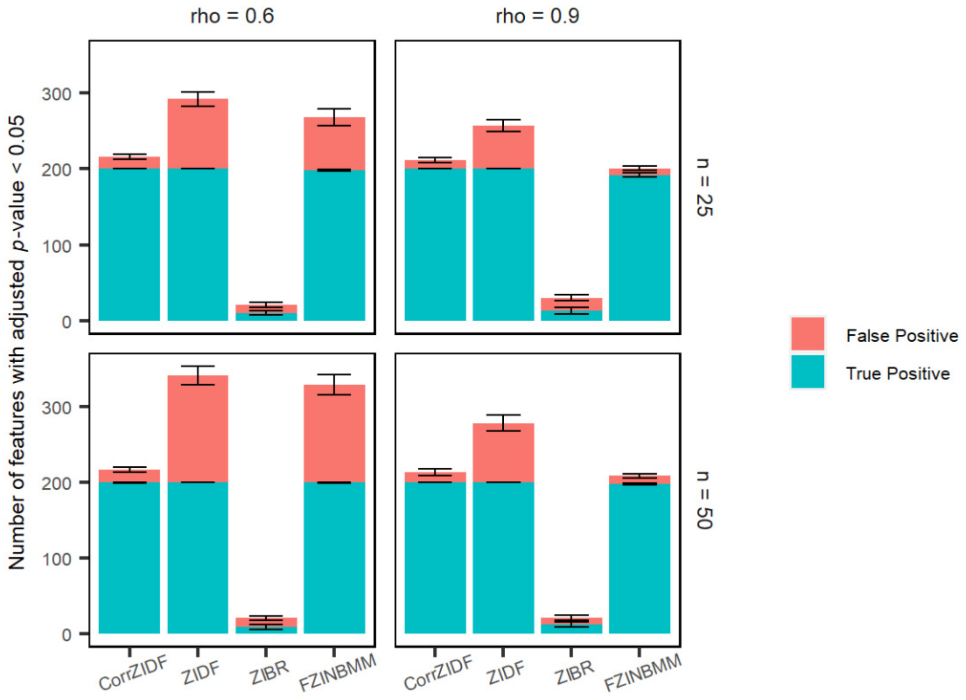

Figure S3. Bar plots of the numbers of detected true and false positives under exchangeable correlation structure. Bar plots in 4 (25/50 subjects per condition with moderate/high correlation) settings based on 20 replicated simulations with 1000 features (including 200 DAFs) after adjusting multiple comparisons by the BH procedure. Each bar represents the total number of features that are detected as statistically significant post BH adjustment, and the short error bars represent the standard deviation from 20 replications. The true number of DAFs in the simulation is 200.

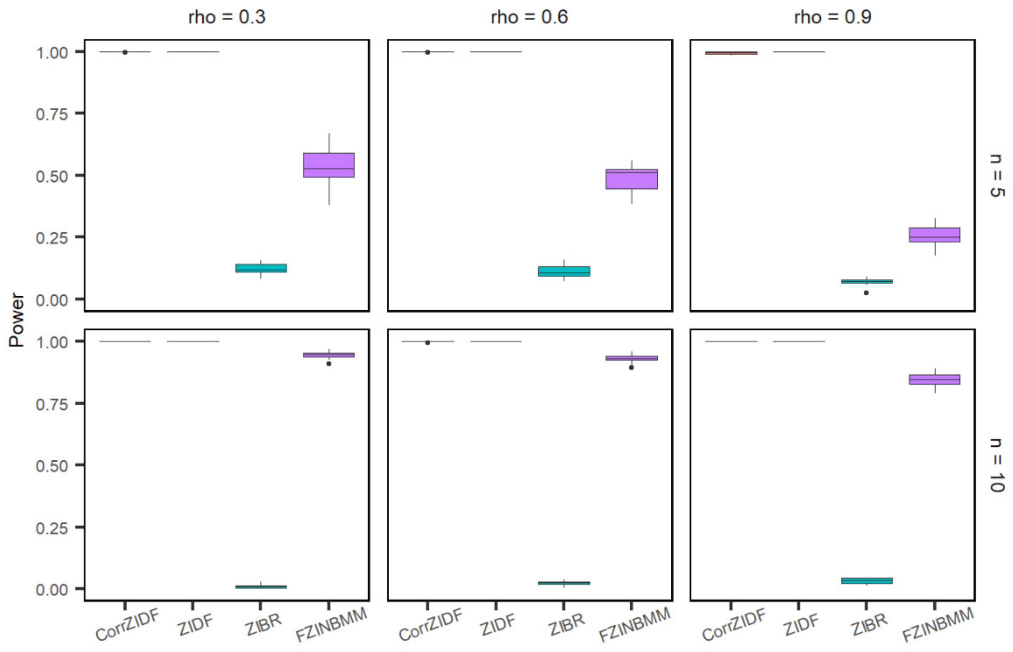

Figure S4. Boxplots of the power for small number of subjects. Boxplots in 6 (5/10 subjects per condition with low/moderate/high correlation) settings based on 20 replicated simulations with 1000 features (including 200 DAFs) after adjusting multiple comparisons by the BH procedure.

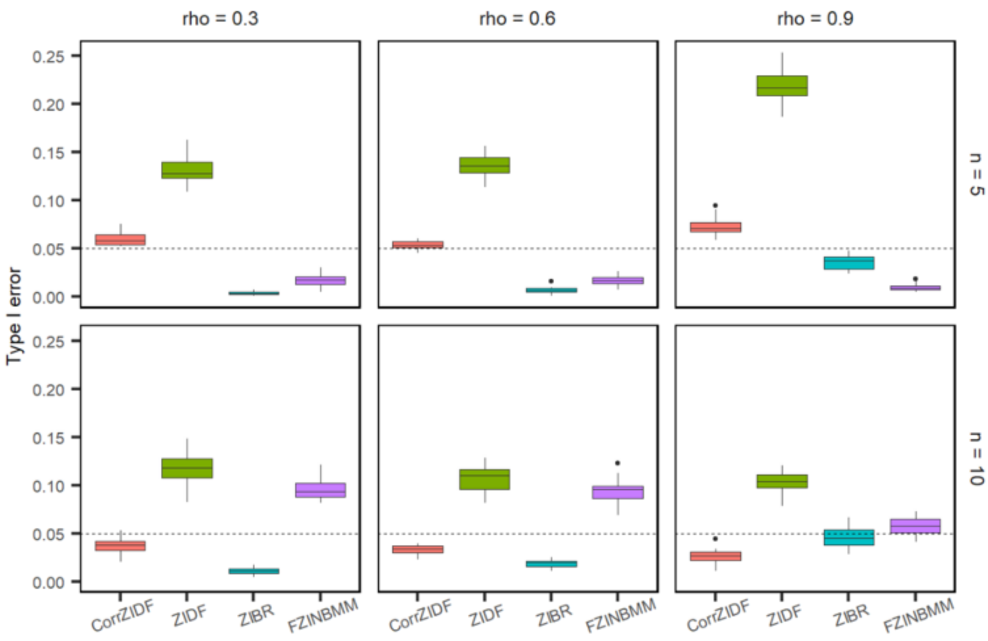

Figure S5. Boxplots of Type I error rates for small number of subjects. Boxplots in 6 (5/10 subjects per condition with low/moderate/high correlation) settings based on 20 replicated simulations with 1000 features (including 200 DAFs) after adjusting multiple comparisons by the BH procedure. The dashed line represents the cutoff of 0.05.

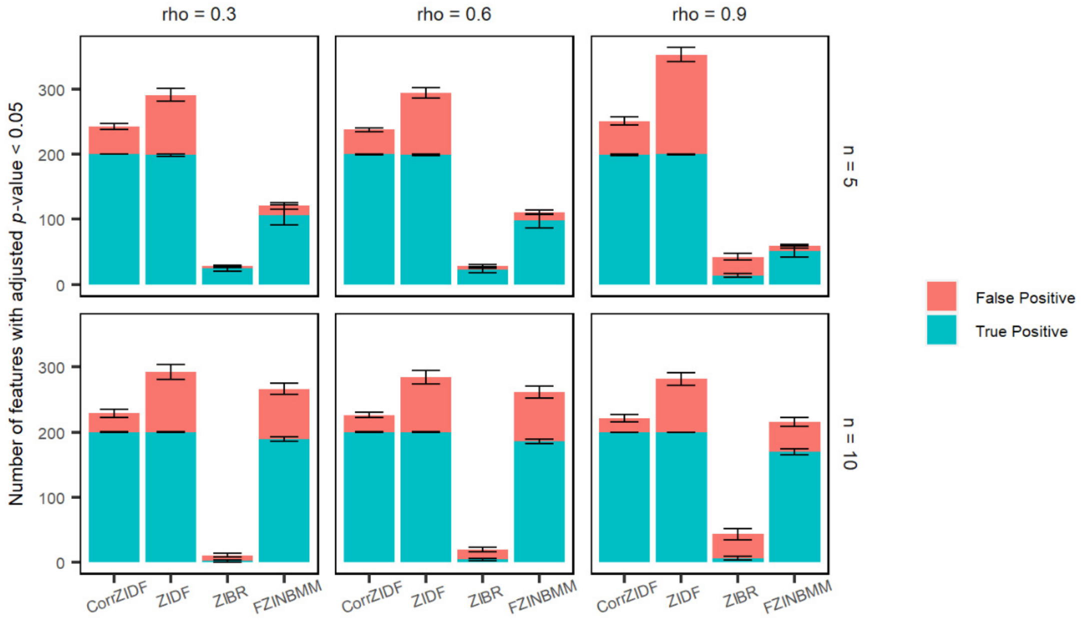

Figure S6. Bar plots of the numbers of detected true and false positives for small number of subjects. Bar plots in 6 (5/10 subjects per condition with low/moderate/high correlation) settings based on 20 replicated simulations with 1000 features (including 200 DAFs) after adjusting multiple comparisons by the BH procedure. Each bar represents the total number of features that are detected as statistically significant post BH adjustment, and the short error bars represent the standard deviation from 20 replications. The true number of DAFs in the simulation is 200.
